# Complement in brain and eye disease: shared mechanisms, convergent pathologies, and common therapeutic opportunities

**DOI:** 10.1007/s00335-026-10199-3

**Published:** 2026-02-03

**Authors:** Jacqui Nimmo, Matthew Bright, Zhizhong Yang, Laura Elisabeth Nicholls, Bryan Paul Morgan, Nikoleta Daskoulidou, Wioleta Milena Zelek

**Affiliations:** UK Dementia Research Institute Cardiff, School of Medicine, Cardiff University, Cardiff, CF235NQ UK

## Abstract

The brain and eye share striking anatomical, physiological, and immunological similarities. Both are protected by specialised vascular barriers, the blood-brain barrier (BBB) and blood-retinal barrier (BRB) respectively, that maintain homeostasis through tightly regulated cellular and molecular mechanisms. Increasing evidence implicates complement dysregulation as a key driver of neurodegeneration in both organs, contributing to disorders such as Alzheimer’s disease (AD), and age-related macular degeneration (AMD). Genetic and molecular studies highlight overlapping mechanisms, with variants in complement regulators influencing susceptibility to both retinal and brain pathologies. Locally synthesised complement components modulate glial activation, vascular integrity, and synaptic remodelling at both sites and, when dysregulated, contribute to chronic inflammation, synapse loss and neuronal degradation. Despite these shared pathways, therapeutic development has progressed asymmetrically; several complement therapeutics are already in the clinic for AMD and other ocular pathologies, while none are yet in use for brain diseases. This is in part a consequence of the accessibility of the eye where complement targeted drugs can be directly delivered and impact on pathology monitored, difficult in the brain. Lessons learned from the eye, including local delivery to overcome challenges of barrier penetration, may help accelerate development of complement therapeutics for the brain. Here we present a brief perspective that integrates current understanding of complement-mediated mechanisms across brain and eye, emphasising clues from convergent pathophysiology and ocular translational successes.

## Introduction

The brain and eye have historically been considered two separate research fields, labelled neurological and ophthalmological research respectively. Yet they are intricately connected, anatomically, physiologically, and immunologically (London et al. 2013). Neurodegeneration in brain and retina, while presenting as clinically distinct entities, share common underlying mechanisms of disease development and progression. This includes dysregulation of complement, a key part of the innate immune system with essential roles in defence against infection and clearance of debris. Complement labels pathogens and debris for phagocytic clearance, recruits and activates phagocytes through the anaphylatoxins C3a and C5a and mediates direct killing of susceptible microbes via the membrane attack complex (MAC). However, inappropriate activation or dysregulation of complement can contribute to tissue injury in many chronic disorders, including retinal and brain pathologies, the focus of this brief perspective. Complement has been implicated in age-related macular degeneration (AMD), glaucoma, multiple sclerosis (MS), ischaemic stroke and Alzheimer’s disease (AD), with emerging roles in other ocular and neurodegenerative diseases (NDDs) (Zelek et al. 2019; Zelek and Tenner 2025). Despite their clinical differences, these disorders share common neuroimmune mechanisms - synapse and neuronal loss, reactive gliosis and vascular impairment – all of which involve complement activation and dysregulation.

**Fig. 1:**
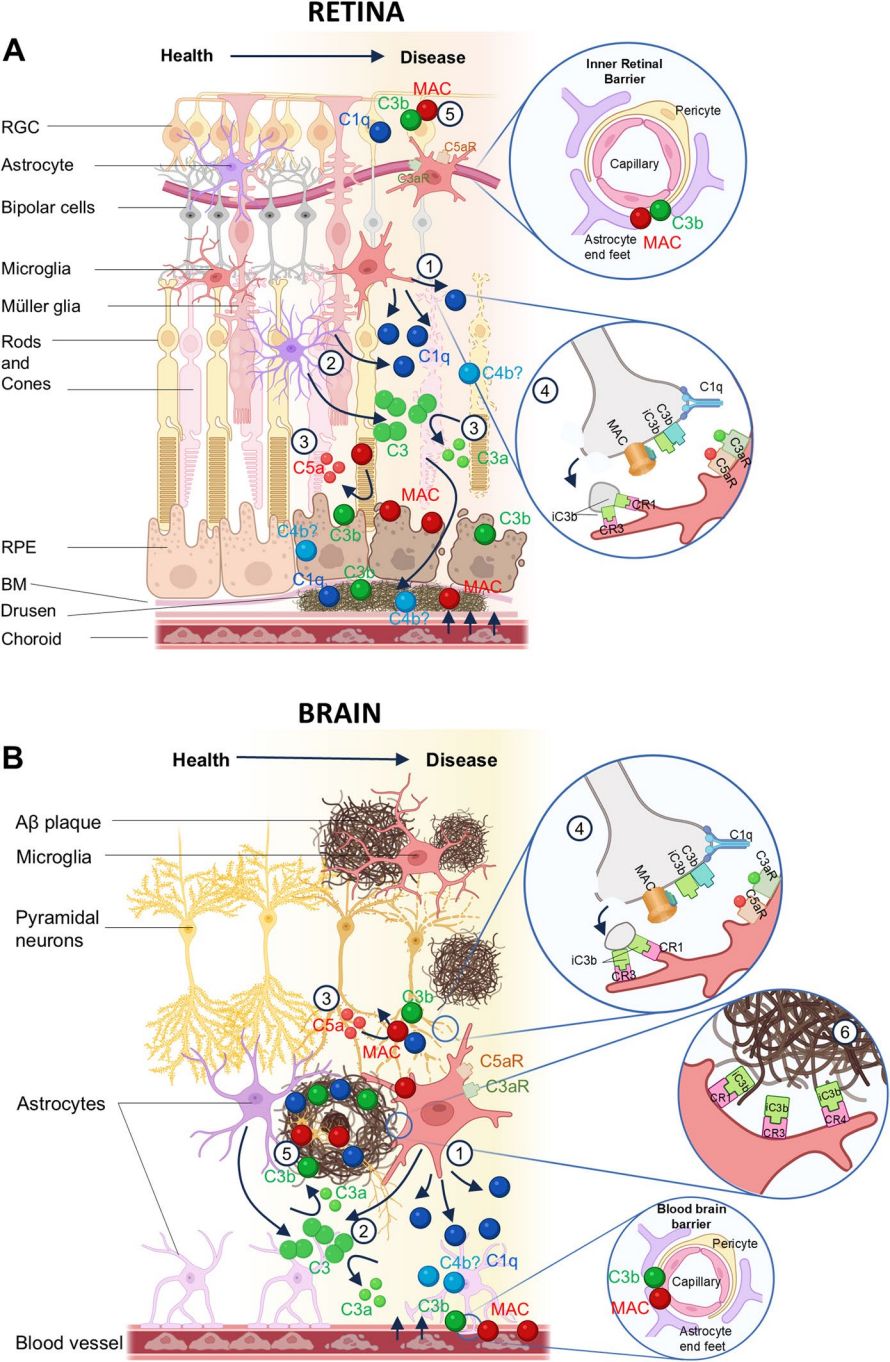
Complement dysregulation in the retina and the brain. **A** Complement is locally synthesised in the retina and upregulated during inflammation. Microglia and Müller glia are the source of C1q in the retina (1, 2) while astrocytes contribute to the local production of C3 (2); the local sources of other complement components in retina are not known. Activation of complement leads to cleavage of C3 and C5 releasing the anaphylatoxins C3a and C5a (3) which recruit glia by binding C3aR and C5aR respectively. C3 cleavage also releases the opsonin C3b which is deposited on drusen, synapses (4) and damaged cell surfaces for phagocytosis by microglia (5). C1q, and complement activation products including C3b, C4b and MAC, accumulate in drusen deposits and promote further inflammation and BRB leakage (4). Formation of MAC within membranes of RPE or RGC contributes to cell damage and barrier leak (5). **B** Complement is locally synthesised in the brain and upregulated during inflammation. Microglia are the sole producers of C1q in the brain (1) and major producers of C3, along with astrocytes and likely other cell types (2). The local sources of other complement components in brain are not known. Activation of complement leads to cleavage of C3 and C5 releasing the anaphylatoxins C3a (2) and C5a (3) which recruit glia by binding C3aR and C5aR (4). C3 cleavage also releases the opsonin C3b which is deposited on amyloid plaques (5), damaged neurons and synapses (4), labelling for phagocytosis by microglia via surface receptors CR1, CR3 and CR4 (4,6). C1q and complement activation products C3b/iC3b, C4b and MAC, accumulate in amyloid plaques and promote further inflammation and glial activation (5). Complement activation at the blood–brain barrier (BBB) contributes to endothelial activation and barrier dysfunction (6)

 Some of the earliest evidence of complement dysregulation in retinal and brain diseases came from immunohistochemistry studies in post-mortem tissue, demonstrating the deposition of complement activation products (C1q, C3 and C5b-9) in hallmark lesions of neurodegeneration; on amyloid beta (Aβ) plaques in AD, on drusen deposits in AMD, and damaged retinal ganglion cells (RGC) in glaucoma (Anderson et al. 2002; Eikelenboom and Stam 1982; Katschke et al. 2018; Tezel et al. 2010; Veerhuis 2011). In retina and brain, the hallmark extracellular proteinaceous deposits, drusen and Aβ plaques respectively, accumulate cell debris and other waste materials, acting as triggers for local complement activation and dysregulation, driving tissue injury and chronic inflammation (Fig. 1 A, B).

**Fig. 2:**
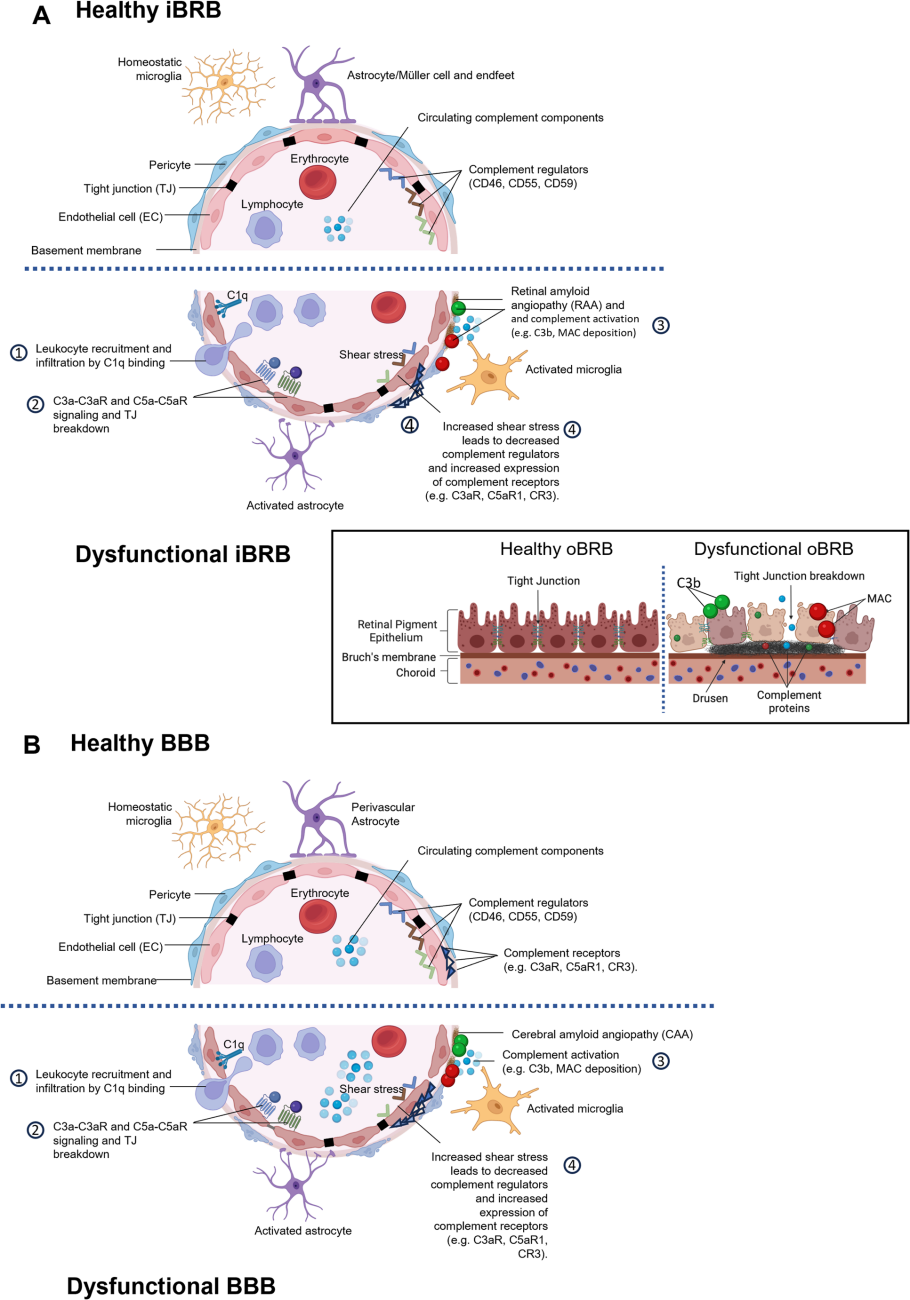
Complement in barrier integrity and vascular dysfunction. **A** Schematic representation of healthy (top) and dysfunctional (bottom) inner blood retinal barrier (iBRB). Leukocyte recruitment and infiltration into the retina are promoted by C1q binding to endothelial cell receptors, signalling upregulation of endothelial adhesion molecules, increasing cell leukocyte adhesion and barrier leakage (1). C3a-C3aR and C5a-C5aR signaling on endothelial cells causes tight junction (TJ) breakdown (2). Retinal amyloid angiopathy (RAA) activates complement to drive barrier damage (3). Increased shear stress causes localised decreased endothelial cell expression of complement regulators CD46, CD55, and CD59, rendering the stressed endothelial cells more vulnerable to complement damage (4). The Insert shows the outer BRB (oBRB), comprising a monolayer of retinal pigment epithelium (RPE) cells with tight junctions between cells that maintain barrier integrity. Complement dysregulation causes MAC-mediated destruction of RPE cells with loss of oBRB integrity. In AMD, deposits of cellular debris known as drusen accumulate between the RPE and Bruch’s membrane and activate complement with C1q, C3b/iC3b, C4b and MAC deposition. **B** Schematic representation of healthy (top) and dysfunctional (bottom) BBB. (1) Leukocyte recruitment and infiltration into the brain are promoted by C1q binding endothelial cell receptors, signalling upregulated expression of adhesion molecules, leukocyte recruitment and barrier leakage. (2) C3a-C3aR and C5a-C5aR signaling on endothelial cells signals tight junction (TJ) breakdown. (3) Accumulation of amyloid within capillary basement membranes in Cerebral amyloid angiopathy (CAA) contributes to local complement activation and vascular damage. (4) Increased shear stress causes localised decrease in expression of complement regulators CD46, CD55, and CD59, on endothelial cells rendering them more vulnerable to complement damage

The retina is an anatomical extension of the brain, connected via the optic nerve and sharing numerous anatomical and physiological characteristics, including comparable barrier systems, vasculature and resident immune cells (London et al. 2013). Both brain and eye are considered immune-privileged, protected by selective barriers, the blood-brain barrier (BBB) and the blood-retinal barrier (BRB), that control communication between the organ and the blood, crucial for homeostasis by restricting peripheral protein and immune cell infiltration (Fig. 2 A, B). The vasculature in the brain and retina is closely similar, both relying on end arteries to supply regions of the organ, making them vulnerable to vascular injury and ischaemic damage. In the retina, as in brain, complement expression and regulation involves multiple cell types, not only resident microglia but also astrocytes, primarily associated with the nerve fibre layer and retinal vasculature, and retina-specific Müller glia, which provide homeostatic and immune-support functions, including local complement production and modulation. Emerging evidence shows that retinal astrocytes adopt reactive states in response to injury, switching on expression of complement proteins, thereby provoking neuronal injury in diseases such as glaucoma and inflammatory optic neuropathies (Gharagozloo et al. 2021; Guttenplan et al. 2021, 2024).

Despite their close connectivity and structural similarities, research on roles of complement in brain and retina have developed in separate silos with relatively few studies exploring brain-retina cross-talk. For example, while the development of anti-complement drugs for use in ocular disease has progressed rapidly, similar approaches for brain diseases remain in their infancy. In AMD, several complement therapies are already in the clinic (e.g., pegcetacoplan, avacincaptad pegol), while in glaucoma, others are in late-stage trials (e.g., ANX007) (Jaffe et al. 2021; Grover et al. 2023; Sun et al. 2023; Khanani et al. 2023; European Medicines Agency 2024a, b; Wykoff et al. 2025), These advances have been made possible by the use of intraocular delivery to subvert barriers; although intraocular injection has enabled effective retinal drug delivery and underpinned the clinical success of therapies such as anti-VEGF mAb (Rosenfeld et al. 2006), the challenges of delivering drugs to the correct anatomical sites within the eye remain. Direct injection into the brain is, for most therapeutic agents, unviable, presenting substantial challenges for therapeutic delivery and target engagement.

We contend that the time has come to integrate the parallel lines of evidence implicating complement in brain and retinal disease into a unified framework that will accelerate translational discovery. The rapid progress of complement therapeutics into the clinic in retinal diseases highlights the delivery constraints for brain but also provides proof-of-concept and a route map to guide strategies for use in brain diseases.

## Genetics implicates complement in brain and eye NDDs

Genetic studies underscore both the shared and distinct aspects of complement dysregulation in brain and retinal diseases. Genome-wide association studies (GWAS) have identified polymorphisms in the genes encoding complement factor H (*CFH*) and complement factor I (*CFI*) as strong risk factors for AMD (Fritsche et al. 2016), while genes encoding the factor H-related protein (*CFHR*) gene cluster, factor B (*CFB*), C2, C3 and C9 have also been implicated (Gehrs et al. 2010; Harris et al. 2012). Indeed, these inherited variants combine in a ‘complotype’, a combination of genetic markers that together impact complement activation and AMD susceptibility (Harris et al. 2012; Paun et al. 2016). The complotype in AMD informed patient stratification for the first anti-complement drug trials in AMD (Fritsche et al. 2016; Paun et al. 2016; Holz et al. 2018; Halawa et al. 2021).

In contrast, AD GWAS have identified risk variants in genes encoding complement receptor 1 (*CR1*) and clusterin (*CLU*) (Lambert et al. 2009), implicating complement in both but suggesting different mechanisms of involvement. Although CR1 and CLU have not been identified as genetic hits in GWAS for AMD, one study found increased clusterin levels in aqueous humor of AMD patients, suggesting that it may play a role in AMD pathology (Rinsky et al. 2021). Interestingly, the *CFH* Y402H polymorphism, well known for its strong association with AMD, has also been associated with AD in Han Chinese populations, suggesting a common regulatory mechanisms in retina and brain (Zhang et al. 2016; Daskoulidou et al. 2025a). Notably, FH, FI and CR1 are all key regulators of the amplification loop (also termed alternative pathway), suggesting that dysregulation of this critical driver of activation underpins the association in both pathologies. Therapeutic agents targeting the amplification loop could thus have applicability in brain and retinal NDDs. CR1, a top-5 GWAS hit in AD, is expressed on microglia with risk variants conferring altered phagocytic capacity (Daskoulidou et al. 2023, 2025b). In the brain, CR1 is expressed by microglia, and AD associated risk variants are linked to altered phagocytic capacity (Daskoulidou et al. 2023, 2025b). By contrast, a clear association between CR1 and ocular disease has not been established. Current evidence suggests that CR1 expression in the retina is low and largely restricted to the retinal pigment epithelium, where experimental overexpression can confer protection against complement-mediated injury (Simmons et al. 2020). However, the physiological relevance of CR1 to retinal complement regulation in vivo remains poorly defined, underscoring important tissue-specific differences in complement receptor utilisation between the brain and retina. Thus, while the genetics implicate complement dysregulation in retina and brain, cellular context and complement expression patterns likely differ.

## Complement biomarkers in NDDs involving brain and eye

Complement proteins and activation products measured in plasma or cerebrospinal fluid (CSF) have been implicated as biomarkers of disease or disease progression in AD (Guo et al. 2025; Hakobyan et al. 2016; Hu et al. 2016; Sandoval et al. 2024; Thambisetty et al. 2011). In particular, plasma and CSF levels of clusterin and C1q have been replicated as markers in numerous studies. A study comparing complement genetics and plasma markers demonstrated that AD-associated variants in *CR1*, *C1S*, and *CFH* genes influenced plasma levels of their encoded proteins, providing a mechanism for the association (Veteleanu et al. 2023). Although not yet adopted into routine testing, these markers have guided understanding of mechanism, advanced AD biomarker discovery and, with genetic data noted above, assisted stratification for clinical trials (Morgan et al. 2019; Veteleanu et al. 2023; Li et al. 2024).

In the eye, aqueous (anterior chamber of the eye) and vitreous (posterior chamber) humour sampling has demonstrated that complement activation products (Ba/Bb, C3a, C3d, C5a, C5b-9), and clusterin accumulated in these compartments may provide supportive evidence of local complement dysregulation (Altay et al. 2019; Mandava et al. 2020; Schick et al. 2017; Rinsky et al. 2021). A recent study comparing complement profile in vitreous and aqueous humour and plasma in GA reported that levels of complement activation markers can differ substantially between these (Hallam et al. 2025), highlighting that aqueous humour is not a consistent proxy for vitreous or retinal complement dysregulation. These findings highlight the need for larger, well-powered studies with harmonised sampling and analytical approaches to robustly define compartment-specific complement signatures in retinal disease..

The eye provides a powerful and non-invasive window into NDD biology. The retina can be examined in exquisite detail using high-resolution methods (OCT, OCT-A, FAF) that enable measurement of retinal thickness, drusen volume, vascular density, and other parameters that can be tracked with precision over time (Rispoli et al. 2023). Amyloid and other disease biomarkers can also be measured and monitored in retina using these methods, providing an under-exploited window into brain pathology (García-Bermúdez et al. 2023; Green et al. 2010; Saidha et al. 2015). While some studies have reported retinal amyloid-β accumulation in AD (Koronyo-Hamaoui et al. 2011; Koronyo et al. 2017), others have failed to confirm these findings (den Haan et al. 2018); hence, the presence of classical amyloid deposits in the AD retina remains controversial. Emerging evidence suggests that retinal neurodegeneration, including thinning of retinal nerve fibre and ganglion cell layers, mirrors brain neurodegeneration in AD, providing a potential non-invasive biomarker (Banna et al. 2024; Jin et al. 2025; Leal-Bernal et al. 2025; London et al. 2013; Vujosevic et al. 2023; Zhang et al. 2021). Ongoing large-scale studies are further exploring the use of retinal scanning to predict dementia risk (Hao et al. 2024; Eppenberger et al. 2024; Edinburgh, 2025). Direct, non-invasive imaging of complement dysregulation has been achieved in other organs using labelled ligands for the complement fragment C3d (Holers 2016; Thurman and Rohrer 2013); similar approaches to imaging complement activation in retina and brain would revolutionise diagnosis and therapeutic strategies.

## Complement expression in glia and gliosis

Because of the presence of barriers preventing the influx of large proteins from plasma in both retina and brain, complement activities, whether homeostatic or pathological, likely rely heavily on local synthesis. Surprisingly little is known about which cells in brain and retina make which complement proteins and under what circumstances. Resident glial cells, given their many roles in brain homeostasis are obvious candidate sources. Microglia are reported to be the principal source of C1q in the brain and retina, while Müller cells provide an additional source of C1q in the retina (Jiao et al. 2018; Stasi et al. 2006). Müller glia and retinal astrocytes are also reported to be the main producers of C3 (Feng et al. 2025; Gharagozloo et al. 2021; Stasi et al. 2006; Tabor et al. 2023). Ocular glia and astrocytes upregulate C1q and C3 production respectively in glaucoma, likely promoting a proinflammatory environment and cell damage (Guttenplan et al. 2020; Guttenplan et al. 2025; Harder et al., 2017; Stasi et al. 2006). Transcriptional data suggest that both microglia and astrocytes contribute to C3 production in brain (Wu et al. 2019; Feng et al. 2025). For other complement proteins, information on local production in brain or eye is sparse. Although a comprehensive atlas of complement protein expression in the human retina is still lacking, this knowledge gap has driven the creation of online repositories of single-cell transcriptomic resources that map cell-type–specific gene expression across human, rodent and organoid retina, providing an important step towards characterising complement biology at cellular level (https://singlecell-eye.org/app/spectacle/).

Receptors for the potent pro-inflammatory complement activation fragments C3a and C5a (C3aR and C5aR respectively) are expressed on microglia in retina and brain and on brain infiltrating macrophages (Tabor et al. 2023). The phagocytic C3 fragment receptors CR1, CR3 and CR4 are present on microglia, upregulated around sites of injury and in disease and play an important role in phagocytosis of pathological protein deposits, synapses and damaged cells (Akiyama and McGeer 1990; Crehan et al. 2013). Indeed, these local deposits of Aβ in AD or drusen in AMD act as ‘hot spots’ for complement activation, driving glial activation, reactive gliosis and further tissue damage, placing complement dysregulation as a central orchestrator of glial activation and neuroinflammation.

## Complement in barrier integrity and vascular dysfunction

The BBB and BRB maintain tissue homeostasis by restricting immune and molecular traffic. Barrier integrity is critical to the healthy functioning of the brain and the eye, while barrier dysfunction contributes to brain and ocular neurodegenerative pathology (Fig. 2 A, B); accumulating evidence indicates that complement plays a pivotal role in barrier disruption in disease. An important distinction in the eye lies in the presence of an outer blood-retinal barrier (oBRB, Fig. 2A-Insert), where the choriocapillaris is separated from the neural retina not by a tight endothelial cell layer, but by Bruch’s membrane, an acellular extracellular matrix that is in continuous contact with the circulation. This configuration renders Bruch’s membrane particularly exposed to systemic complement and susceptible to C3b deposition. Protection of this interface depends almost entirely on acquisition of the soluble complement regulators FH and FHL-1 which, in concert with FI, constitute the principal regulators of complement activation on this acellular surface. Consequently, genetic or functional perturbations affecting these regulators exert a disproportionate effect at the oBRB and are strongly implicated in AMD pathology (Clark et al. 2014; Clark et al. 2015; Clark et al. 2017). The inner BRB (iBRB) and BBB are structurally similar, comprising tightly joined specialised endothelial cells with associated pericytes and glia; these cells express receptors and regulators for multiple complement proteins. Endothelial cells are continuously exposed to circulating complement components that can affect function. For example, C1q binds endothelial cell receptors and upregulates the expression of adhesion molecules (E-selectin, ICAM-1 and VCAM-1), promoting leukocyte recruitment and infiltration into the brain (Lozada et al. 1995). Relevance to disease is provided by the observation that administration of C1 inhibitor (C1-INH) reduced endothelial expression of adhesion molecules and BBB leakage, and improved outcomes in a mouse cerebral ischaemia model (Heydenreich et al. 2012; Storini et al. 2005). Endothelial cells also express receptors for C3a (C3aR) with C3a-C3aR signalling modulating endothelial tight junction integrity and permeability (Bhatia et al. 2021; Wu et al. 2016); C3aR-deficient mice showed improved vascular and cognitive outcomes in models of vascular dementia (Bhatia et al. 2022). C5a-C5aR signalling similarly compromises BBB integrity, both in vivo and in vitro (Flierl et al. 2009; Jacob et al. 2010; Mahajan et al. 2015).

In AD, barrier integrity is also compromised by the accumulation of Aβ around smooth muscles cells (SMCs) of cerebral arteries, known as cerebral amyloid angiopathy (CAA). While mechanisms of SMC damage are not well characterised, recent reports have demonstrated complement deposition in arteries of CAA cases in which the presence of terminal complement complexes (TCC) was associated with microhaemorrhages (Hondius et al. 2023; Matsuo et al. 2017). Consistent with this, treatment of AD mouse models with clusterin, a regulator of terminal complement activation, protected from vascular damage (Hu et al. 2023). Similarly, the terminal pathway regulator CD59 conferred protection to vascular SMCs from complement attack, suggesting a role of the terminal complement pathway and MAC formation in CAA and vascular damage (Whinnery et al. 2024).

Increased shear stress occurs in the vasculature at specific areas of turbulence and in association with vascular ageing and hypertension; this causes localised decreased endothelial cell expression of complement regulators, including CD46, CD55 and CD59, rendering these areas susceptible to complement damage (Cui et al. 2017; Inafuku et al. 2018; Urbich et al. 2000). In the brain and retina, complement dysregulation at these sites mediates vascular dysfunction and barrier disruption, contributing to pathology in dementias and ocular disease. Indeed, C3a-C3aR signalling has been implicated in a mouse model of VCID (vascular contribution to cognitive impairment and dementia) where C3aR knockout reduced neuroinflammation and rescued cognition (Bhatia et al. 2022).Together, these studies demonstrate that the interplay between vascular stress, endothelial complement regulator and receptor expression and complement activation represents a critical point of convergence of barrier dysfunction in ocular and brain neurodegeneration.

## Complement-mediated pruning and synapse loss

Despite differences in structure and function of neurons between brain and retina, the synapses that connect and mediate communication between them are conserved; degeneration of synapses is the key hallmark of both brain and ocular NDDs (Fig. 1A, B). The association of complement tagging with elimination of synapses was first described in a seminal study of synaptic pruning during development in mice; synapses destined for elimination were labelled with complement protein C1q, while mice deficient in C1q or C3 exhibited reduced synaptic pruning and elimination (Stevens et al. 2007). This early discovery prompted the hypothesis that complement was a driver of synaptic elimination in development and the suggestion that complement might also underlie synapse loss in NDDs. This latter suggestion was tested in AD mouse models; as shown in development, deficiency of either C1q or C3 prevented synapse loss in AD mice, while genetic deletion or pharmacological inhibition of the complement terminal pathway offered additional protection, implicating the MAC in synapse elimination (Carpanini et al. 2022; Zelek et al. 2024, 2025).

Synapse loss is also a key feature of retinal development and neurodegeneration in ocular NDDs and complement is strongly implicated. In development, C1q tags RGCs and synapses destined for elimination leading to C3 fragment opsonisation and clearance by retinal microglia (Anderson et al. 2019). In models of glaucoma and AMD, mice deficient in either C1q or C3 showed reduced synapse loss and preservation of retinal integrity (Jha et al. 2011; Williams et al. 2016; Yednock et al. 2022). In a study of the impact of complement on dendritic and synaptic architecture in glaucoma models, C1q deficiency in a genetic model (D2.*C1qa*^−/−^ mouse) and C1 inhibition in a model triggered by raising intraocular pressure (rat bead model), both showed that absence or inhibition of C1 is sufficient to preserve dendritic and synaptic architecture (Williams et al. 2016).

Although the importance of complement in synapse elimination in development and disease in retina and brain is now broadly accepted, there remain several unknowns, including the identity of the ligands for C1q binding expressed on synapses destined for elimination, the extent to which synapse opsonisation and microglial phagocytosis drive elimination and the contributions of the complement activation products C3a, C5a and MAC to synapse loss. Resolution of these knowledge gaps will inform better targeting of anti-complement drugs in the future.

## Complement therapeutics: what’s approved in the eye – and why not (yet) used in brain?

As is clear from the preceding sections, complement dysregulation is a shared driver of neurodegeneration in retina and brain; however, the therapeutic landscape is strikingly asymmetric. Complement inhibitors are already in the clinic for AMD and in trials for other ocular NDDs. In 2023, the FDA approved the use of the C3 inhibitory peptide pegcetacoplan (Syfovre) and the C5-blocking pegylated aptamer avacincaptad pegol (Izervay), delivered intravitreally (IVT), for the geographic atrophy (GA) subset of AMD, marking the dawn of a new era for complement therapeutics in NDDs. Approval was granted based on large, well-designed trials demonstrating reduction of GA lesions by pegcetacoplan (~ 22% (NCT03525600, NCT03525613), (Wykoff et al. 2025) and avacincaptad pegol **(**~ 32% (NCT04435366, NCT02686658) (Jaffe et al. 2021; Khanani et al. 2023), the first demonstration that complement inhibition can slow progression of a chronic NDD. These successes in AMD have spurred a surge of new drugs in trials, and new disease targets, including IVT-delivered anti-C1q Fab (ANX007) in glaucoma (NCT03488550; NCT04188015) (Grover et al. 2023; Sun et al. 2023) and GA (NCT04656561, NCT06510816), a factor B antisense oligonucleotide drug (IONIS-FB-LRx) in GA (NCT03815825; NCT03446144), the orally active factor D inhibitor Danicopan in GA (NCT05019521), and subretinal adenoviral delivery of factor I in GA (NCT03846193, NCT05481827, NCT04566445, NCT04437368). Not all approaches have progressed successfully; the anti-C3 monoclonal antibody (mAb) NGM621 failed to meet its primary endpoint in a Phase 2 trial (NCT04643886), showing only a modest, non-significant slowing of GA progression (~ 6%) (NGM Bio 2023) and the IONIS-FB-LRx program was discontinued in 2024. AAVCAGsCD59 (JNJ-1887; HMR59), an adenovirus designed to locally deliver CD59 and inhibit MAC formation in GA, showed only a mild, non-significant slowing of GA progression in phase 2a trials (NCT03144999; Heier et al. 2024), perhaps reflecting the limited potency of soluble CD59 as an inhibitor, but has nevertheless progressed to an ongoing phase 2b trial (NCT05811351). Emerging next generation single administration gene therapies are expanding the complement-therapeutic toolset in GA: notably, CTx001 (AAV2-mini-CR1) recently received Investigational New Drug approval by FDA and is advancing through early-phase studies (Rathi et al. 2025) (Table 1).

**Table 1:** Complement therapeutics in retinal and brain diseases

Target	Drug	Modality	Disease	Delivery	Clinical Trial Identifiers	Stage	Outcome/Status
RETINA							
C3	Pegcetacoplan (Syfovre)	Cyclic peptide	GA secondary to, AMD; GA	IVT	NCT03525600; NCT03525613	Approved (FDA)	↓ lesion progression
C5	Avacincaptad pegol (Izervay)	RNA aptamer (oligonucleotide)	GA, MD; Dry AMD, GA	IVT	NCT04435366; NCT02686658	Approved (FDA)	↓ lesion progression
C1q	ANX007	mAb	Open Angle Glaucoma	IVT	NCT03488550; NCT04188015	Phase I Completed	safety and tolerability
C1q	ANX007	mAb	GA	IVT	NCT0465656; NCT06510816	Phase II Completed/ Phase III Ongoing	Lack of efficacy/ Active, not recruiting
Factor B	IONIS-FB-LRx	Antisense oligonucleotide	GA secondary to AMD	Subcutaneous injection	NCT03815825; NCT03446144	Phase II Completed/Phase II Withdrawn	Results pending/ No participants enrolled
Factor B	Iptacopan	Small molecule	AMD	Oral tablet	NCT05230537	Phase II Ongoing	Active, not recruiting
Factor D	Danicopan	Small molecule	GA secondary to AMD	Oral tablet	NCT05019521	Phase II Terminated	Lack of efficacy
Factor D	Lampalizumab	mAb	GA secondary to AMD,	IVT	NCT02247479; NCT02247531	Phase III Terminated	Lack of efficacy
C3	NGM621	mAb	Dry AMD, RD, GA	IVT	NCT04643886	Phase II Terminated	No clinical benefit
Factor I	GT005	Gene therapy, (AVV2)	Dry AMD, AMD, GA ED, MA	Subretinal injection	NCT05481827; NCT03846193; NCT04566445; NCT04437368	Phase II Enrolling by invitation/Phase I/II Terminated/Phase II Terminated/	Safety follow-up only (no dosing)/Lack of efficacy/Futility/
C3b/C4b	CTx001	Gene therapy (AVV2; mini CR1)	GA secondary to AMD	Subretinal injection	IND cleared (NCT pending)	Phase I entry	Dosing expected Q1 2026
MAC	JNJ-1887 (HMR59)	Gene therapy (AAV2; sCD59)	Dry AMD, GA	Subretinal injection	NCT03144999; NCT05811351	Phase I Completed/Phase IIb ongoing	Safety and tolerability/Active not recruiting

BRAIN							
C1q	ANX005	mAb	HD, ALS	IV	NCT04514367; NCT04569435	Phase IIa Completed	Safety, PK/PD; exploratory efficacy
C5	Ravulizumab	mAb	ALS	IV	NCT04248465	Phase III Terminated	Lack of efficacy
C5	Zilucoplan	Cyclic peptide	ALS	IV	NCT04436497	Phase II/III Completed	No clinical benefit
C5	Ravulizumab (Ultomiris)	mAb	NMOSD	IV	NCT04201262	Approved (FDA)	Prevents relapses

*AMD* Age-related macular degeneration, *ALS* Amyotrophic lateral sclerosis, *ED* Eye degeneration, *AVV2* Adeno-associated virus serotype 2, *GA* Geographic atrophy, *HD* Huntington’s disease, *IVT* Intravitreal; IV Intravenous mAb: monoclonal antibody, *MAC* Membrane attack complex, *MD* Macular degeneration, *NMOSD* Neuromyelitis optica spectrum disorder, *RD* Retinal degeneration

In contrast to the remarkable progress in retinal diseases, complement therapeutics in the brain remain in early-stage development. ANX005, an anti-C1q mAb, has been trialled in Huntington’s disease (HD), met endpoints in early trials and is currently in an open-label Phase 2a HD trial (NCT04514367) with patients stratified for complement dysregulation. ANX005 is also in a Phase 2a amyotrophic lateral sclerosis (ALS) trial (NCT04569435). Notably, trials of a C5-blocking (mAb), ravulizumab (NCT04248465) (Genge et al. 2023) and a C5-blocking peptide, zilucoplan (NCT04436497), in ALS were stopped for futility (Paganoni et al. 2025), highlighting the importance of trial design and patient stratification.

Why have complement therapeutics progressed rapidly to the clinic in ocular NDDs but not yet in NDDs impacting the brain? There are two main reasons. First, *accessibility*; IVT delivery is relatively simple, reliable, repeatable, and achieves pharmacologically relevant levels at the site of pathology with limited systemic exposure. In contrast, intrathecal (IT) delivery requires injection into the CSF or the brain itself, difficult, potentially dangerous and not suitable for frequent dosing. Local delivery is further limited by the large brain volume and the need for sustained, safe target engagement across the organ. Delivery from the periphery to the brain is restricted by the BBB, meaning that currently available systemic complement inhibitors cannot (usually) access the brain, highlighting the need for brain-penetrant drugs. Second, *trackability*; trials in GA and other retinal NDDs can be monitored precisely by retinal imaging as described above, while brain NDDs evolve slowly, are highly heterogeneous and difficult to monitor, requiring complex endpoints and better biomarkers.

The success of anti-complement drugs in trials of retinal NDDs provide proof-of-concept for the use of complement therapeutics in brain NDDs but also highlight the limitations of current approaches. Both pegcetacoplan and avacincaptad pegol slowed progression of GA but did not reverse vision loss and caused side effects including macular neovascularisation (~ 12% of cases) and, rarely, severe retinal vasculitis. Currently, these drugs cost, respectively, 26,000 per patient per year, a cost-benefit profile considered inadequate for approval by the European Medicines Agency (European Medicines Agency 2024a, b). These problems around efficacy, safety, ease of delivery and cost are precisely the hurdles we must anticipate in developing drugs for brain NDDs. The first hurdle to be cleared is that of delivery.

**Fig. 3:**
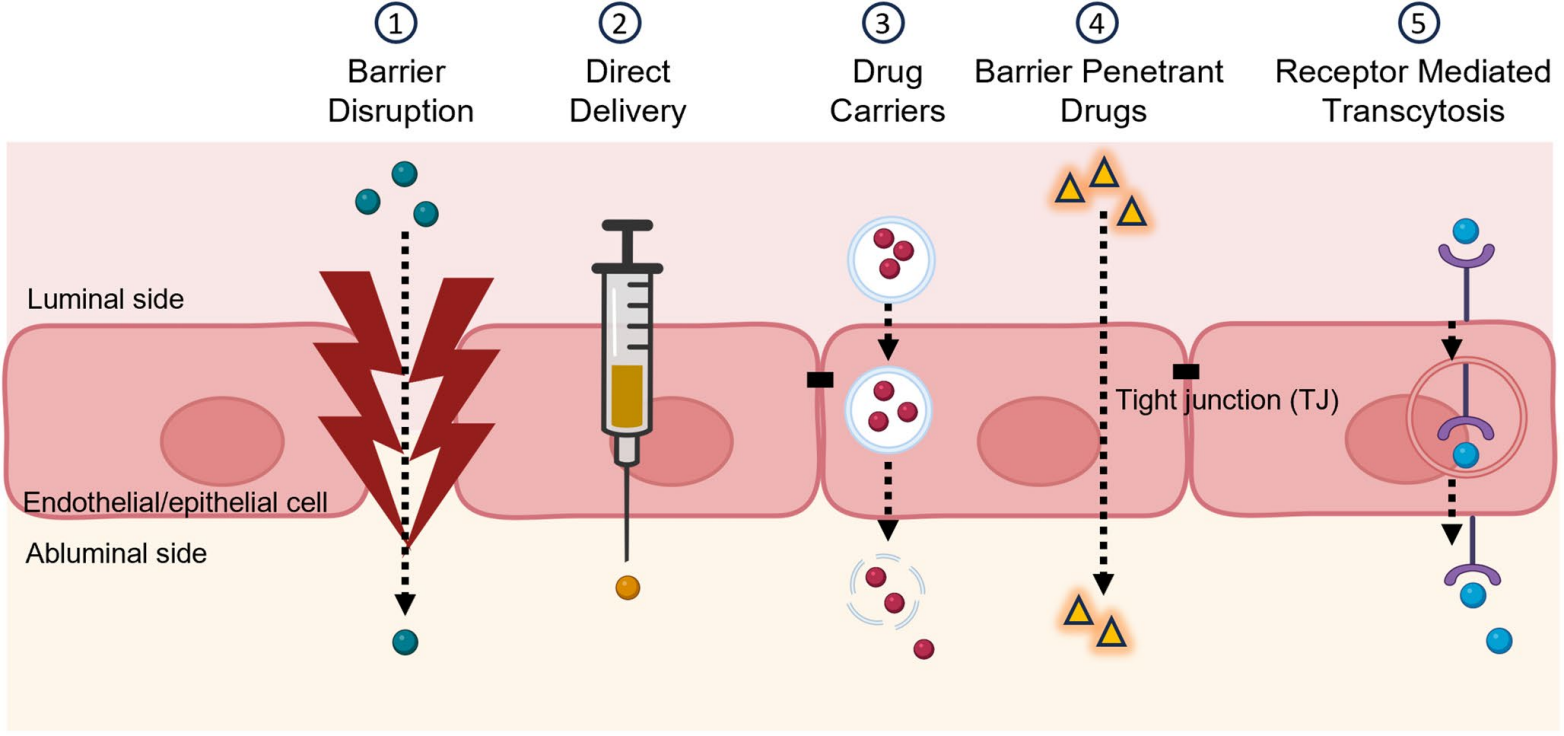
Routes of drug delivery across the blood-brain barrier (BBB) or blood-retinal barrier (BRB). Several approaches exist to deliver drugs across the BBB or BRB. This includes disruption of the barrier via localised ultrasound (1); direct delivery into the eye (intravitreal injection) or brain (intrathecal or intracerebral injection) (2); packaging drugs into vesicles to help cross the barrier (3); developing small lipophilic molecule that can enter the brain freely (4) or through receptor mediated transcytosis (5). Figure modified from Zelek and Morgan 2022.

In ocular diseases, IVT enables drug delivery directly closer to the site of injury; comparable approaches in brain, IT or intraparenchymal delivery, are highly invasive and not feasible for long-term treatment of chronic disease. Systemic or oral delivery are the preferred routes, but the BBB restricts entry of most large molecules (Pardridge 2020). Receptor-mediated transcytosis (RMT) has emerged as a credible solution, exploiting the endogenous brain endothelial transporters, for example, the transferrin receptor (TfR), to increase brain uptake of mAbs and other large molecules (Bonvicini et al. 2025; Chew et al. 2023; Haqqani et al. 2024; Khoury et al. 2025; Wells et al. 2025). (Fig. 3). Several BBB-shuttle platforms are already in use, including for delivery of enzyme replacement therapies for rare inherited diseases such as Hunter syndrome (Okuyama et al. 2021) and mAbs targeting amyloid and tau for AD therapy (Grimm et al. 2023; Yang 2025). Preclinical studies have demonstrated that these delivery shuttles are directly applicable to complement therapeutic cargos. A C7-blocking mAb coupled to TfR-binding shuttle was successfully delivered to the brain in App^NL−G−F^ AD mice, reduced complement dysregulation, rescued synapse loss and improved cognition (Zelek et al. 2025).

## Conclusions and future directions

Complement dysregulation is a driver of neurodegeneration in retina and brain and a critical pathway in NDDs affecting these organs. Shared target structures (synapses, neurons) dictate shared mechanisms and pathways. Disease initiators may differ between organs and diseases - aggregates of extracellular Aβ or tau in brain parenchyma in AD, extracellular Drusen deposits within the retinal pigment epithelium (RPE) and basement membrane (BM) in AMD and damaged retinal neurons secondary to increased intraocular pressure in glaucoma; however, recruited components and downstream consequences are strikingly conserved. Clinical trials in ocular NDDs have proven that locally delivered complement inhibition can slows disease progression, altering the course of a chronic neurodegenerative disease. Lessons from these trials – successful and unsuccessful – should inform strategies for progression to treatment of brain NDDs. For example, the trials to date have demonstrated heterogeneity of response, underlining the need for biomarker-driven stratification and patient selection.

While the case for translating these treatment approaches to brain NDDs is made, the principal barriers remain logistical (delivery), statistical (endpoints), and biological (timing). With imaging and genotype/biomarker-informed trial design, we are now positioned to stratify patients with precision, selecting the right patients, choosing the right anti-complement drugs and treating at the right stage of disease evolution. We need to develop and optimise better methods to measure neurodegeneration, inflammation and complement dysregulation in biofluids and brain, and explore further ways of using the retina as an accessible window on disease in the brain. With better biomarkers and new barrier-penetrant complement therapeutics in the pipeline, we anticipate the broad application of these new agents, given systemically or orally, for effective and safe therapy of eye and brain NDDs.

## Data Availability

No datasets were generated or analysed during the current study.
